# Identification of novel arsenic resistance genes in yeast

**DOI:** 10.1002/mbo3.1284

**Published:** 2022-04-30

**Authors:** Esin Isik, Çiğdem Balkan, Vivien Karl, Hüseyin Çağlar Karakaya, Sansan Hua, Sebastien Rauch, Markus J. Tamás, Ahmet Koc

**Affiliations:** ^1^ Department of Molecular Biology and Genetics Izmir Institute of Technology Izmir Turkey; ^2^ Department of Chemistry and Molecular Biology University of Gothenburg Gothenburg Sweden; ^3^ Water Environment Technology, Department of Architecture and Civil Engineering Chalmers University of Technology Gothenburg Sweden; ^4^ Department of Genetics, School of Medicine Inonu University Malatya Turkey

**Keywords:** arsenate, arsenite, Pho86, Tul1, Ugp1, Vba3

## Abstract

Arsenic is a toxic metalloid that affects human health by causing numerous diseases and by being used in the treatment of acute promyelocytic leukemia. *Saccharomyces cerevisiae* (budding yeast) has been extensively utilized to elucidate the molecular mechanisms underlying arsenic toxicity and resistance in eukaryotes. In this study, we applied a genomic DNA overexpression strategy to identify yeast genes that provide arsenic resistance in wild‐type and arsenic‐sensitive *S. cerevisiae* cells. In addition to known arsenic‐related genes, our genetic screen revealed novel genes, including *PHO86, VBA3, UGP1*, and *TUL1*, whose overexpression conferred resistance. To gain insights into possible resistance mechanisms, we addressed the contribution of these genes to cell growth, intracellular arsenic, and protein aggregation during arsenate exposure. Overexpression of *PHO86* resulted in higher cellular arsenic levels but no additional effect on protein aggregation, indicating that these cells efficiently protect their intracellular environment. *VBA3* overexpression caused resistance despite higher intracellular arsenic and protein aggregation levels. Overexpression of *UGP1* led to lower intracellular arsenic and protein aggregation levels while *TUL1* overexpression had no impact on intracellular arsenic or protein aggregation levels. Thus, the identified genes appear to confer arsenic resistance through distinct mechanisms but the molecular details remain to be elucidated.

## Graphical abstract

In this study, we utilized a genomic DNA overexpression strategy to identify yeast genes that provide arsenic resistance in *Saccharomyces cerevisiae* cells. The contribution of selected genes, including *PHO86*, *VBA3*, *UGP1*, and *TUL1*to cell growth, intracellular arsenic content, and protein aggregation levels during exposure to arsenate was analyzed to provide insights into resistance mechanisms. Our data suggest that the identified genes confer arsenic resistance through distinct mechanisms.

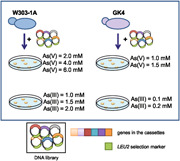

## 1 INTRODUCTION

The metalloid arsenic is abundantly present in the environment mainly as pentavalent arsenate [As(V)] and trivalent arsenite [As(III)]. Both forms of arsenic are toxic and chronic exposure causes cancers of the skin, lung, bladder, kidney, and liver, and is associated with various neurodegenerative disorders. Exposure occurs mainly via toxic waste or through the consumption of contaminated drinking water and food. Chronic arsenic exposure is a significant public health concern with hundreds of millions of persons worldwide estimated to be affected (Clemens & Ma, 2016; Naujokas et al., 2013).

At the cellular level, arsenic's toxic and carcinogenic effects may be caused by the inactivation of specific enzymes, induction of oxidative stress, inhibition of DNA repair systems, and deregulation of cell proliferation. As(V) resembles phosphate and may replace phosphate in some reactions and interfere with ATP synthesis. As(III) has a high affinity for sulfhydryl groups and can bind to cysteine residues in proteins, thereby altering their folding and activity. Additionally, As(III) may cause oxidative damage to proteins, lipids, and DNA (Hughes et al., 2011; Shen et al., 2013; Tamás et al., 2014). Despite its toxicity, arsenic has a long history of usage as a therapeutic agent and is currently used to treat acute promyelocytic leukemia. The disease‐causing and therapeutic effects of arsenicals have spurred research into the molecular mechanisms that underlie their toxicity and the mechanisms that cells use to develop resistance. Most of these studies have focused on model organisms, such as bacteria and the eukaryote model *Saccharomyces cerevisiae* (budding yeast) (Garbinski et al., 2019; Rosen & Tamás, 2010; Slyemi & Bonnefoy, 2012; Wysocki & Tamás, 2010).

Arsenic exerts its toxic effects inside cells. Therefore, transport proteins that allow the influx of arsenic or mediate its export are central players of toxicity and resistance. In budding yeast, like in most cells, As(V) is taken up by phosphate transporters (Pho84 in *S. cerevisiae*) (Bun‐ya et al., 1996; Yompakdee et al., 1996), while As(III) is taken up by aquaglyceroporins (Fps1) (Wysocki et al., 2001) and hexose transporters (Liu et al., 2004). Intracellular As(V) is reduced to As(III) by the arsenate reductase Acr2 (also called Arr2) (Mukhopadhyay & Rosen, 1998; Mukhopadhyay et al., 2000) followed by As(III) export from the cytosol through the plasma membrane arsenite efflux protein Acr3 (also called Arr3) (Wysocki et al., 1997) or sequestration into the vacuole in form of the glutathione conjugate As(GS)_3_ catalyzed by the ABC (ATP‐binding cassette) transporter Ycf1 (Ghosh et al., 1999). These transport proteins are regulated at the transcriptional and posttranslational levels during As(III) stress. For instance, the expression of *ACR2* (*ARR2*) and *ACR3* (*ARR3*) is regulated by the arsenic‐sensing transcription factor Yap8 (also called Acr1 or Arr1) (Kumar et al., 2016; Menezes et al., 2004; Wysocki et al., 2004), *YCF1* expression is regulated by Yap1 while expression of glutathione biosynthesis‐related genes involves the transcription factors Yap1 and Met4 (Thorsen et al., 2007; Wysocki et al., 2004). The activity of the yeast aquaglyceroporin Fps1 is regulated by the kinases Hog1 and Slt2 (Ahmadpour et al., 2016; Lee & Levin, 2018; Thorsen et al., 2006) as well as the Rgc1 and Rgc2 proteins (Beese et al., 2009) whereas hexose transporters are degraded in response to As(III), probably as a way to gain resistance (Jochem et al., 2019). Another cellular defense strategy employed by most cells is arsenic chelation. *S. cerevisiae* increases the production of the tri‐peptide glutathione in response to As(III) exposure for intracellular and extracellular chelation and detoxification (Talemi et al., 2014; Thorsen et al., 2007, 2012). Since arsenic induces widespread misfolding and aggregation of newly synthesized proteins (Ibstedt et al., 2014; Jacobson et al., 2012), decreased protein synthesis and proteasomal degradation of misfolded/aggregated proteins represent additional mechanisms used by cells to evade arsenic toxicity (Andersson et al., 2021; Guerra‐Moreno et al., 2015; Jacobson et al., 2012). A number of other genes and pathways with a putative role in resistance or toxicity have been identified in comprehensive genome‐wide phenotypic screens using the yeast genome‐wide deletion collection (Haugen et al., 2004; Jin et al., 2008; Jo et al., 2009; Johnson et al., 2016; Pan et al., 2010; Thorsen et al., 2009; Zhou et al., 2009) or via transcriptomic or proteomic approaches (da Silva et al., 2018; Haugen et al., 2004; Hosiner et al., 2009; Jin et al., 2008; Jochem et al., 2019; Matia‐Gonzalez & Rodriguez‐Gabriel, 2011; Thorsen et al., 2009). In this current study, we performed a high‐copy genomic DNA (gDNA) library screening in yeast and identified genes whose overexpression confer arsenic resistance.

## 2 MATERIALS AND METHODS

### 2.1 Yeast strains, growth conditions, plasmids, and gDNA library screening

The *S. cerevisiae* strains used in this study are listed in Table A1. Deletion mutants were purchased from EUROSCARF and the *HSP104*‐GFP strain was purchased from Invitrogen. Haploid and diploid deletion mutants are respectively in BY4741 and BY4743 backgrounds, lacking the corresponding gene but carrying the kanamycin‐resistant gene instead. YPAD (1% yeast extract, 2% peptone, 0.04% adenine, 2% glucose) was used as a rich medium and SD (synthetic dextrose with 0.67% YNB (yeast nitrogen base) without amino acids, 2% glucose and supplemented with auxotrophic requirements) was used as a selective medium for regular cultivation of yeast cells. Sodium arsenate, Na_2_HAsO_4_ (Cat. No: A6756), and sodium arsenite, NaAsO_2_ (Cat. No: S7400), were purchased from Sigma‐Aldrich.

High copy yeast genomic DNA library (AB320 genomic DNA library in YEp13 plasmid in *Escherichia coli* obtained from the American Type Culture Collection ATCC [No: 37323]) was introduced into yeast cells by transformation for subsequent genomic library screening. The transformation was carried out by the standard LiAc method.

**Figure 1:**
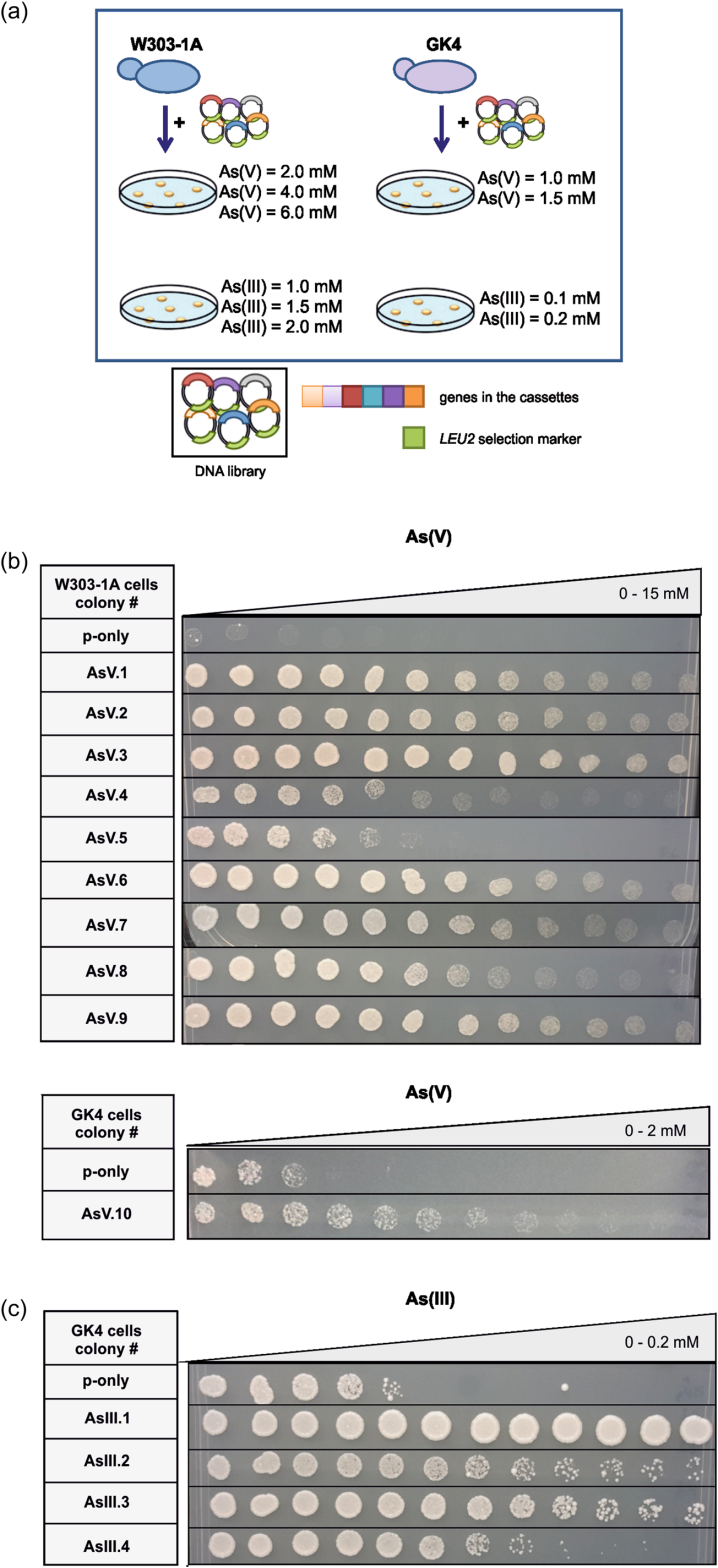
Screening of a genomic library for arsenic resistance in yeast and identified hits. (a) Cartoon of the screening setup and arsenic concentrations used. Growth of yeast gDNA transformants on gradient plates containing (b) arsenate [As(V)] and (c) arsenite [As(III)]. Growth was scored after incubating the cells for 3–5 days at 30°C. P‐only represents the empty vector containing cells.

The setup of the screen and the arsenic concentrations used are illustrated in Figure 1a. The plasmids present in the resistant colonies were isolated by a commercial miniprep kit (GeneJET Plasmid Miniprep Kit; Thermo‐Molecular Biology) after treating the yeast cells with lyticase (5 U/ml) for 30 min. The isolated plasmids were amplified in *JM109 E. coli* cells and sequenced with vector‐specific sequencing primers using DNA sequencer ABI3130xl with ABI PRISM sequencing analysis v5.1 program at IzTech Biotechnology Center.

### 2.2 Cloning of selected candidate genes

Selected candidate genes were cloned from the yeast Open Reading Frame (ORF) collection (in *E. coli*, YSC3868) into the pAG425GPD‐ccdB plasmid by the Gateway Cloning System. Since the *PHO86* gene was not available in the ORF collection, it was amplified by PCR using a pair of Gateway‐compatible primers and then cloned into pAG425GPD‐ccdB. The correct cloning of the candidate genes was confirmed by restriction enzyme analysis and sequencing with vector‐specific primers.

### 2.3 Growth assay on gradient plates

A gradient plate was prepared with two different layers of agar media to produce a continuous concentration gradient of the metalloid in a Petri dish, as previously reported (Szybalski & Bryson, 1952). Initially, 50 ml of agar medium without metalloid was poured as a lower layer onto a square Petri dish (120 mm × 120 mm) and then tilted at an angle to cover the complete bottom surface of the plate. After solidification, the plate was positioned horizontally and 50 ml of medium with the corresponding arsenical at the desired concentration was poured on top of the solid medium. The arsenicals in the upper layer diffused toward the lower layer in proportion to the heights of the layers and, because of this diffusion, a uniform concentration gradient was established increasing from 0 mM on one side to the highest arsenic concentration on the other side. An overnight culture of yeast cells in the corresponding growth medium was diluted and grown to the logarithmic phase. Then, 5 μl of the cells with an OD_600_ value of 0.02 were spotted on the gradient plates starting from one end of the plate to the other end. The plates were incubated at 30°C for 3–5 days and the growth of the cells was recorded.

### 2.4 Arsenic measurements

Plasmids overexpressing the indicated genes were introduced into yeast cells (BY4741). The transformants were grown to exponential phase in a YNB medium containing 2% glucose and subsequently, cells were either left untreated or exposed to 1 mM As(V) for 1 h. Samples were collected before and after As(V) exposure and washed twice in ice‐cold water. The cell pellet was then resuspended in water, boiled for 10 min, and centrifuged to collect the supernatant. To measure arsenic efflux, cells were first incubated with 1 mM As(V) for 1 h to allow arsenic accumulation, then the cells were washed with As(V)‐free medium, and samples were taken for intracellular arsenic determination. The arsenic content of each sample was determined by inductively coupled plasma‐mass spectrometry (ICP‐MS) following a 20‐fold dilution with ultrapure water from a GenPure water purification system (Thermo Fisher Scientific; Barnstead, resistivity 18.2 MΩ cm) and acidification to 1% volume HNO_3_ (Sharlau, HNO_3_, 65% for trace analysis) before the analysis. The analysis was performed on an ICAP Q ICP‐MS (Thermo Fisher Scientific) with an SC‐FAST automated sample introduction system (Elemental Scientific). Arsenic was measured at *m*/*z* = 75 and the instrument was operated in the Kinetic Energy Discrimination (KED) mode with He as the collision gas to remove potential interference from ArCl^+^ at *m*/*z* = 75. Calibration was performed using a set of arsenic standards with concentrations up to 500 µg L^−1^. A solution of 1 µg L^−1^ indium was continuously injected for internal standardization. The detection limit is estimated to be 0.1 µg As L^−1^.

### 2.5 Protein aggregation assay

Plasmids were introduced into yeast cells (BY4741) harboring a genomic copy of the Hsp104‐GFP fusion protein (Huh et al., 2003). The transformants were grown to mid‐log phase in YNB medium and exposed to 2 mM As(V). Cells were collected at the indicated time points, fixed with formaldehyde for 30 min at room temperature, and washed with phosphate‐buffered saline (PBS). The GFP signals were observed using a Zeiss Axiovert 200 M (Carl Zeiss MicroImaging) fluorescence microscope equipped with Plan‐Apochromat 1.40 objectives and appropriate fluorescence light filter sets. Images were taken with a digital camera (AxioCamMR3) and processed with Zeiss Zen software. To quantify protein aggregation, the fraction of cells with aggregates/Hsp104–GFP foci was determined by visual inspection of 110–230 cells per strain and time point using Image J software.

### 2.6 Localization of Pho84‐GFP and Pho86‐GFP

The plasmids p*PHO84‐GFP* (EB0666) and p*PHO86‐GFP* (EB0667) (Lau et al., 2000) were introduced into BY4741 cells. Overnight cultures of the transformants were diluted in fresh liquid media and then treated with 0.5 mM As(V) for 1 h. Cells were transferred to microscope slides and analyzed with a fluorescence microscope.

## 3 RESULTS

### 3.1 High‐copy genomic library screening for genes conferring resistance to arsenicals

**Figure A1:**
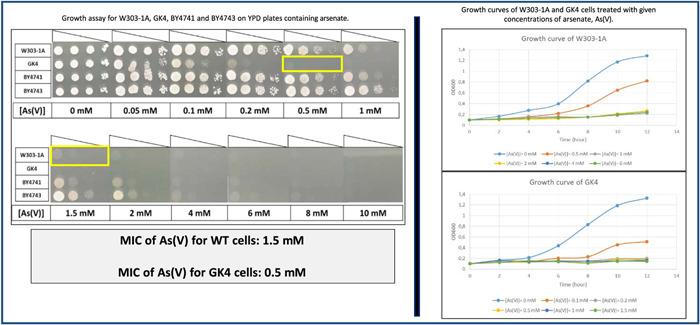
Determining the minimum inhibitory concentration (MIC) for arsenate [As(V)] in the indicated yeast strains.

**Figure A2:**
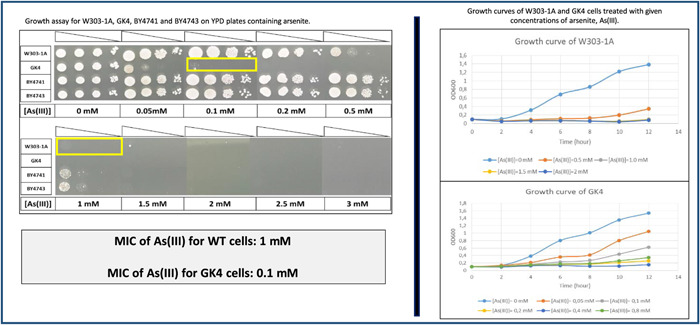
Determining the minimum inhibitory concentration (MIC) for arsenite [As(III)] in the indicated yeast strains.

To identify novel arsenic resistance genes, we screened a high‐copy gDNA library in two different yeast backgrounds: W303−1A (wild‐type) and the arsenic‐sensitive GK4 (Δ*acr1* Δ*acr2* Δ*acr3*) strains (Figure 1a; Table A1). Initially, we performed growth assays on plates to assess minimum inhibitory concentration (MIC) values of As(III) and As(V). 1.5 and 0.5 mM of As(V) completely inhibited the growth of W303‐1A and GK4, respectively, while the MIC of As(III) was determined to be 1.0 mM and 0.1 mM for W303‐1A and GK4 cells, respectively (Figures A1 and A2). After determining the MICs, the yeast genome library was inserted into the W303‐1A and GK4 strains, and the transformants were screened on plates containing As(V) or As(III) at lethal concentrations (>MIC) (Figure 1a). Surviving colonies were selected for further experiments.

**Figure A3:**
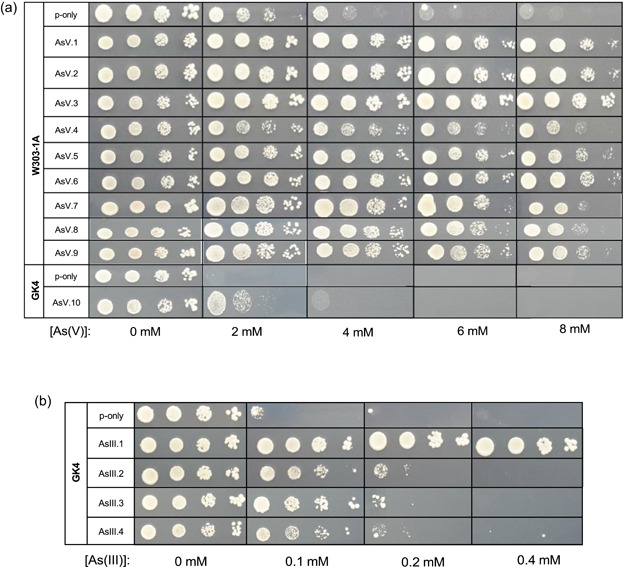
Growth of yeast gDNA transformants on plates containing given concentrations of (a) arsenate [As(V)] and (b) arsenite [As(III)]. Growth was scored after incubating the cells for 5 days at 30°C. P‐only represents the empty vector.

We first confirmed the arsenic resistance of the surviving colonies from the library screen by growth assay on gradient plates. Subsequently, plasmids present in the resistant colonies were isolated and amplified in *E. coli*. The amplified plasmids were inserted back into the corresponding yeast strain. Growth assays were carried out to confirm that the resistance observed in the original transformants was due to the presence of the plasmids. Consequently, ten colonies for As(V) (9 for W303‐1A and 1 for GK4) and four colonies for As(III) (GK4) resistance were selected (Figures 1b,c and A3). The plasmids from these colonies were sequenced with vector‐specific primers. Subsequent bioinformatics analysis revealed the genomic regions present in these plasmids (Table 1). The genomic library used in this study was constructed by ligation of the fragments generated by Sau3AI partial digestion (Nasmyth & Reed, 1980), thus most of the cassettes contained multiple genes, except for one colony (AsIII.3, Table 1).

**Table 1:** The genomic regions of the plasmids from arsenate‐/arsenite‐resistant colonies

**Colony #**	**Chromosome**	**Region**	**Candidate genes** a
AsV.1	X	188,721 to 195,519 bp	*NCA3*, *PHO86*, *YJL118W*, *RPE1*, *ALB1*, *MTC1*
AsV.2	X	191,189 to 196,530 bp	*ASF1*, *NCA3*, *PHO86*, *YJL118W*
AsV.3	XVI	929,988 to 941,100 bp	*ARR1*, *ARR2*, *ARR3*, *SGE1*, *YPR196W*
AsV.4	X	368,844 to 373,815 bp	*TUL1*, *UGP1*, *AIM26*
AsV.5	X	188,958 to 196,530 bp	*ASF1*, *NCA3*, *PHO86*, *YJL118W*, *RPE1*, *ALB1*, *MTC1*
AsV.6	X	191,207 to 196,530 bp	*NCA3*, *PHO86*, *YJL118W*
AsV.7	X	189,834 to 204,440 bp	*ASF1*, *NCA3*, *PHO86*, *YJL118W*, *RPE1*, *ALB1*
AsV.8	X	190,300 to 196,690 bp	*ASF1*, *NCA3*, *PHO86*, *YJL118W*, *RPE1*
AsV.9	X	188,958 to 194,280 bp	*NCA3*, *PHO86*, *YJL118W*, *RPE1*, *ALB1*
AsV.10	X	186,335 to 193,712 bp	*PHO86*, *YJL118W*, *RPE1*, *ALB1*, *MTC1*, *LSM1*
AsIII.1	XVI	932,323 to 943,489 bp	*ARR1*, *ARR2*, *ARR3*, *SGE1*
AsIII.2	XIII	250,986 to 256,885 bp	*GIS4*, *YAP1*, *ERG6*, *MRPL39*
AsIII.3	III	9676 to 14,296 bp	*VBA3*
AsIII.4	XIII	250,986 to 256,885 bp	*GIS4*, *YAP1*, *ERG6*, *MRPL39*

^a^ Note that *ARR1* is also called *ACR1/YAP8*; *ARR2* is also called *ACR2*; *ARR3* is also called *ACR3*.

### 3.2 Genes whose overexpression confers arsenate resistance

Ten different As(V) resistant colonies were obtained: eight of those contained genes from the same genomic fragment encompassing the genes *ASF1, NCA3, PHO86, YJL118W, RPE1, ALB1, MTC1, LSM1*, while two of the colonies contained distinct expression cassettes with genes *ARR1, ARR2, ARR3, SGE1, YPR196W* and *TUL1, UGP1, AIM26*, respectively (Table 1). The presence of the known arsenic resistance gene cluster, consisting of *ARR1/ACR1/YAP8*, *ARR2/ACR2*, and *ARR3/ACR3* (Bobrowicz et al., 1997), in one of the colonies (AsV.3) validates the gDNA library screening approach. The other genes in that cassette (*SGE1*, *YPR196W*) have no known role in arsenic resistance. Likewise, none of the genes in cassette AsV.4 (*AIM26*, *UGP1, TUL1*) have previously been associated with arsenic resistance. *AIM26* encodes a protein of unknown function, *UGP1* encodes a UDP‐glucose pyrophosphorylase, and *TUL1* encodes a Golgi‐localized RING‐finger ubiquitin ligase. The remaining eight cassettes comprised overlapping gene fragments from Chromosome X with *PHO86* and *YJL118W* genes present in all cassettes. Pho86 is an endoplasmic reticulum (ER)‐resident protein that is required for ER exit of the inorganic phosphate transporter Pho84 (Lau et al., 2000). Both *PHO86* and *PHO84* have been implicated in As(V) resistance; Pho84 is responsible for the internalization of arsenate into yeast cells (Shen et al., 2012), and deletion of either *PHO84* or *PHO86* increases As(V) resistance (Bun‐ya et al., 1996). *YJL118W* encodes a protein of unknown function with no known role in arsenic resistance. Deletions of some of the other genes in these cassettes were found in genome‐wide screens to cause As(III) sensitivity, including in *ASF1*, involved in nucleosome assembly and DNA repair (Pan et al., 2010), and *RPE1* encoding a d‐ribulose‐5‐phosphate 3‐epimerase (Haugen et al., 2004; Thorsen et al., 2009). The genes *NCA3, ALB1, MTC1*, and *LSM1* have not been implicated in arsenic resistance previously.

Although we performed the gDNA library screening in both W303‐1A and GK4 cells, we obtained As(III)‐resistant colonies only in the GK4 background. Out of the four colonies, one contained the known arsenic resistance genes *ARR1/ACR1/YAP8*, *ARR2/ACR2*, and *ARR3/ACR3* (Bobrowicz et al., 1997), validating our approach. Two colonies had identical cassettes containing the *GIS4*, *YAP1*, *ERG6*, and *MRPL39* genes. Among these, deletion of *YAP1* and *ERG6* sensitizes cells to As(III) (Haugen et al., 2004; Pan et al., 2010; Thorsen et al., 2009; Wysocki et al., 2004), and *YAP1* overexpression improves arsenic resistance in cells lacking the vacuolar ABC transporter Ycf1 (Bouganim et al., 2001). *GIS4* and *MRPL39* have not been implicated in arsenic resistance previously. One colony contained a single gene in the cassette, *VBA3*, encoding a vacuolar basic amino acid transporter with no previous function in arsenic resistance.

### 3.3 Cloning and overexpression of selected genes

**Figure 2:**
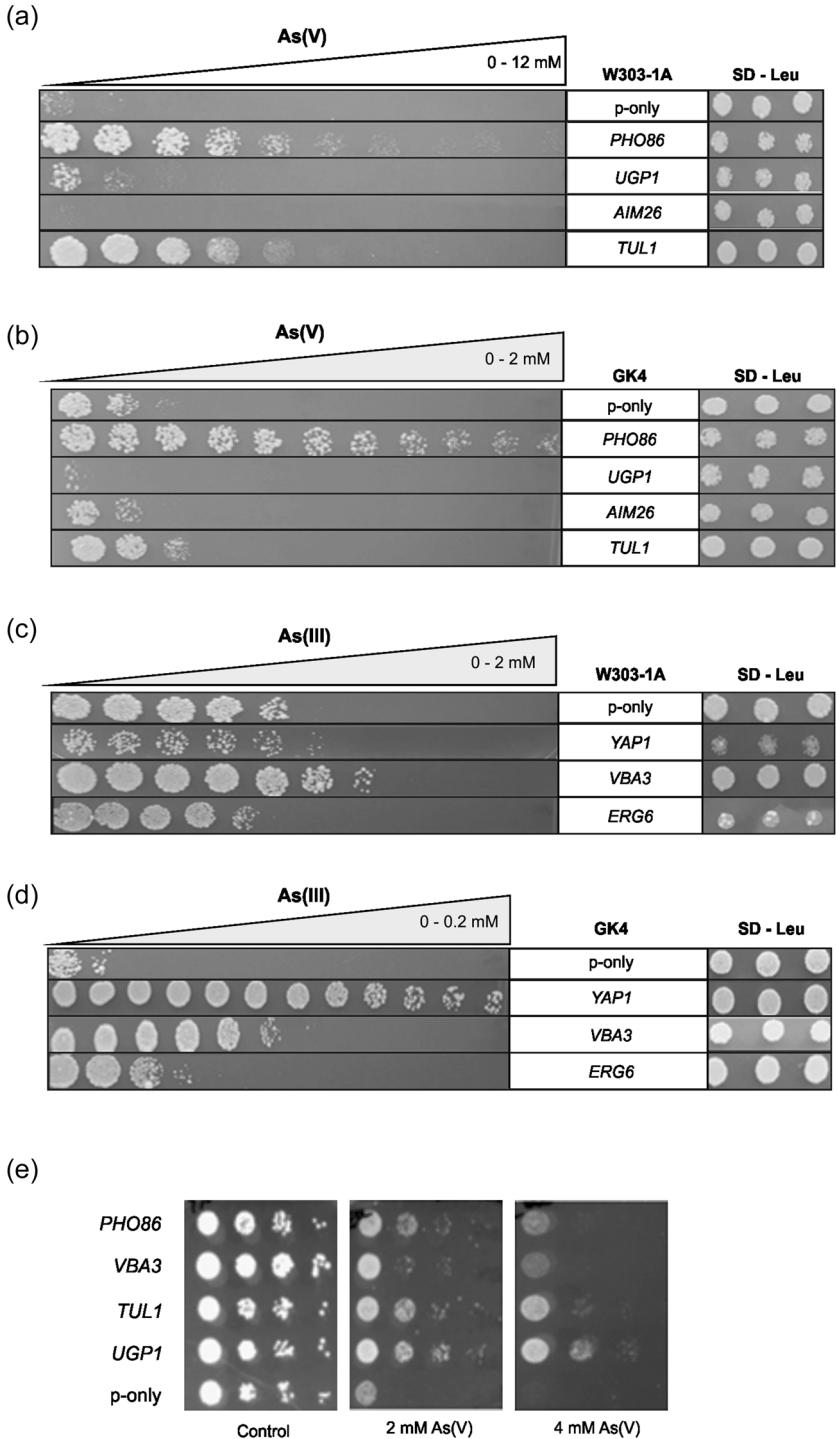
Growth of yeast cells (wild‐type W303‐1A and arsenic‐sensitive GK4) overexpressing individual candidate genes in the presence of arsenate [As(V)] (a,b) and arsenite [As(III)] (c,d). Growth was scored on gradient plates after incubating the cells for 3–5 days at 30°C. Growth of BY4741 cells overexpressing individual candidate genes in the presence of As(V) (e). Growth on plates containing the given As(V) concentrations was scored after incubating the cells for 2–3 days at 30°C. P‐only represents the empty vector containing cells.

**Figure A4:**
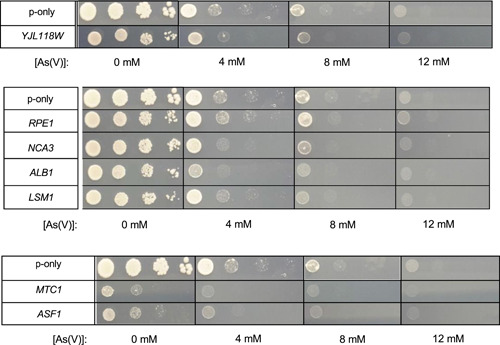
Growth of yeast (W303‐1A) cells expressing individual candidate genes in the absence and presence of given concentrations of As(V). Growth was scored after incubating the cells for 3–5 days at 30°C. P‐only represents the empty vector.

We next cloned and expressed selected genes from a high‐copy expression plasmid (pAG425GPD) in W303‐1A and GK4 cells. The growth of yeast cells overexpressing these candidate genes was then tested in the presence of As(V) and As(III). The different cassettes with *PHO86* and *YJL118W* contained eight genes in total. Overexpression of the *PHO86* gene provided resistance to As(V) in both wild‐type W303‐1A and arsenic‐sensitive GK4 cells (Figure 2a,b) but did not improve As(III) resistance, indicating an As(V)‐specific role for Pho86. In contrast, individual overexpression of *YJL118W, NCA3, RPE1, ALB1*, or *LSM1* did not confer resistance to As(III) or As(V) (Figure A4). Cells overexpressing *MTC1* or *ASF1* grew poorly both in the absence and presence of As(V) (Figure A4), suggesting that these genes have a negative impact on growth and resistance. Of the three genes in cassette AsV.4, individual overexpression of *TUL1* and *UGP1* improved As(V) resistance of W303‐1A cells but not of arsenic‐sensitive GK4 cells, while overexpression of *AIM26* did not improve growth in either strain background (Figure 2a,b). Thus, of the three genes in colony AsV.4, both *TUL1* and *UGP1* contributed to the observed resistance whereas *AIM26* did not. Overexpression of the tested As(III)‐resistant genes (*VBA3*, *YAP1, ERG6*) improved As(III) resistance of GK4 cells, while in W303‐1A cells, only *VBA3* overexpression conferred As(III) resistance (Figures 2c,d). *VBA3* overexpression also resulted in As(V) resistance in the GK4 background. For our follow‐up experiments, we decided to focus on selected genes and their roles in As(V) resistance in a strain background (BY4741), which could be used for all further experiments. We confirmed the As(V)‐resistant phenotype of wild‐type cells of the BY4741 background overexpressing *PHO86*, *VBA3*, *TUL1*, and *UGP1* (Figure 2e). Although all four genes conferred As(V) resistance upon overexpression, we noted that their potency was different in the BY4741 background compared to W303‐1A. W303‐1A is generally more sensitive to various stress conditions than BY4741, and this is also the case for As(III) and As(V) (see Figures A1 and A2). Thus, the degree of the benefit provided by overexpression of individual genes appears to depend on the strain background.

### 3.4 Intracellular arsenic levels

**Figure 3:**
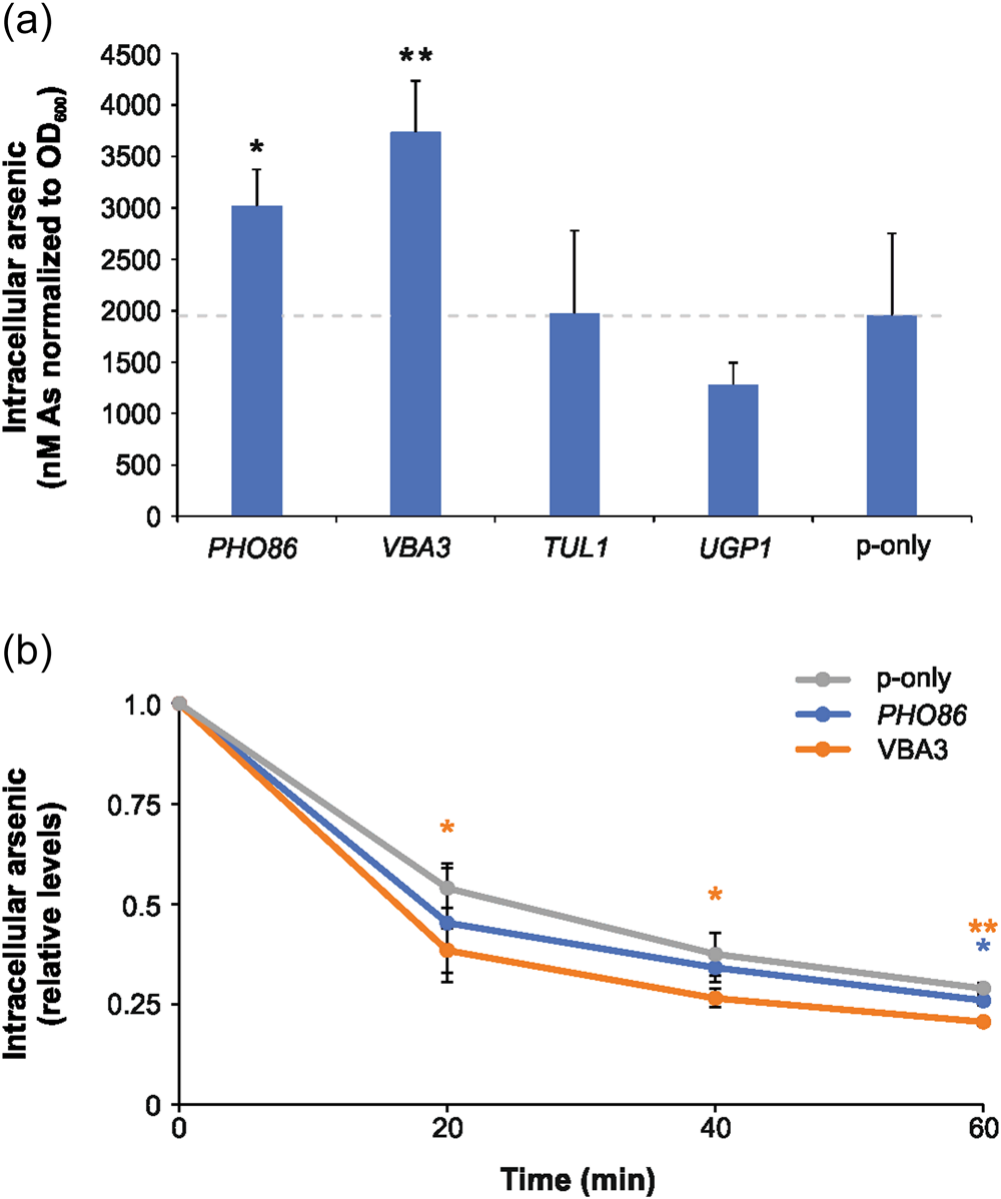
Intracellular arsenic concentrations of BY4741 cells overexpressing the *PHO86, VBA3, TUL1*, and *UGP1* genes or the empty vector control (p‐only). (a) Arsenic accumulation. Intracellular arsenic levels were measured in untreated cells and cells exposed to 1 mM As(V) for 1 h. Values represent averages from six independent biological replicates (*n* = 6) and the error bars represent the standard deviation. (b) Arsenic efflux. Cells were incubated with 1 mM As(V) for 1 h, then washed with As(V)‐free medium, and samples were taken at the indicated time points for intracellular arsenic determination. The intracellular arsenic levels measured before the wash were set to 1 to allow comparisons between cells overexpressing different genes. The values represent averages from three independent biological replicates (*n* = 3) and the error bars represent the standard deviation. Statistical analyses were performed by Student's *t* test, and significant differences are indicated; *p < 0.05 and **p < 0.005.

Since arsenic exerts its toxic effects through intracellular targets, we reasoned that some of the identified genes might affect arsenic uptake, efflux, or intracellular sequestration pathways. To address this, we exposed BY4741 cells to 1 mM As(V) for 1 h and determined intracellular arsenic levels (Figure 3a). There was a significant increase in intracellular arsenic in cells overexpressing *PHO86* (*p* = 0.01) and *VBA3* (*p* = 0.001) compared to cells carrying the empty plasmid (p‐only). Thus, *PHO86* and *VBA3* overexpressing cells are resistant despite elevated intracellular arsenic levels. Intracellular arsenic levels were unchanged in cells overexpressing *TUL1* (*p* = 0.97) and slightly lower in cells overexpressing *UGP1* (borderline of statistical significance with *p* = 0.08). Thus, enhanced resistance of *UGP1* overexpressing cells might be related to less intracellular arsenic.

Chelation and vacuolar sequestration are major arsenic detoxification pathways in yeast (Rosen & Tamás, 2010; Wysocki & Tamás, 2010) and we have previously shown that cells with efficient vacuolar sequestration capacity retain more intracellular arsenic upon removal of extracellular arsenic (Talemi et al., 2014). We therefore asked whether intracellular arsenic in *PHO86* and *VBA3* overexpressing cells is retained or whether it can leave cells upon removal of extracellular As(V). For this, we first incubated cells with 1 mM As(V) for 1 h, then washed the cells with As(V)‐free medium and measured how fast the cells exported the intracellular arsenic. Cells overexpressing *VBA3* exported arsenic more efficiently than wild‐type cells carrying the empty plasmid while the difference between *PHO86* overexpressing and control cells was largely nonsignificant (Figure 3b). These data argue against a model in which *PHO86* and *VBA3* overexpressing cells have a more efficient vacuolar sequestration capacity than wild‐type cells.

### 3.5 Protein aggregation analyses

**Figure 4:**
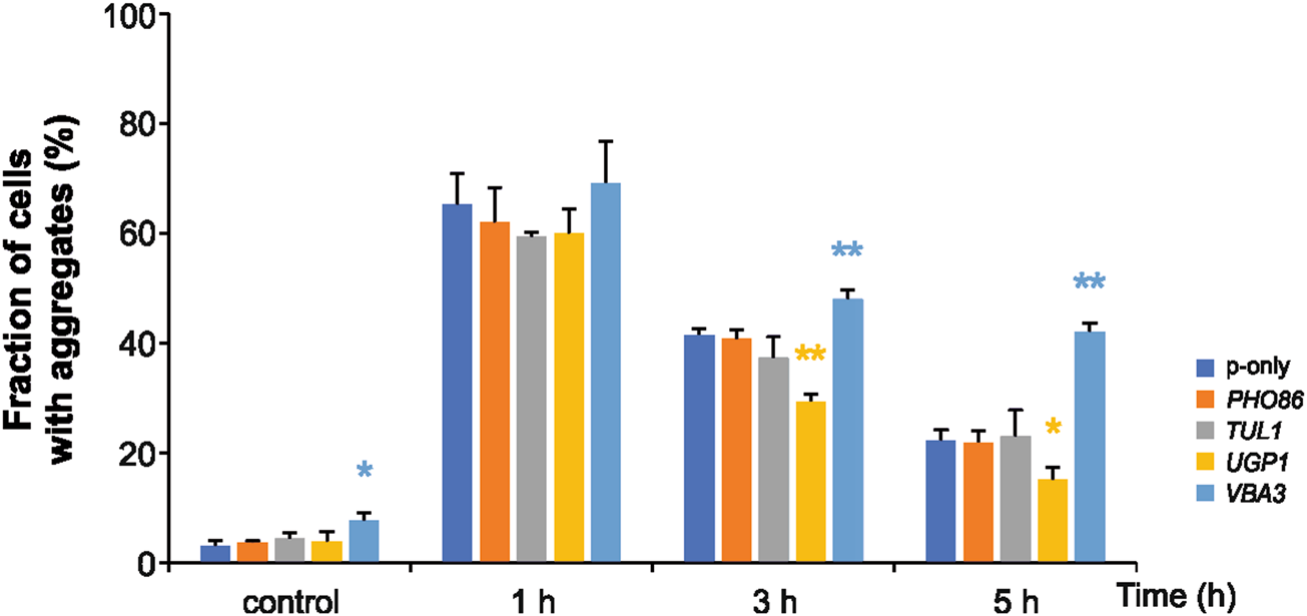
Protein aggregation levels of cells overexpressing the *PHO86, VBA3, TUL1*, and *UGP1* genes. P‐only represents the empty vector containing cells. Hsp104–GFP localization was monitored in BY4741 cells by fluorescence microscopy before and after exposure to 2 mM As(V). The fractions of cells containing aggregates/Hsp104‐GFP foci were determined by visual inspection of 144–282 cells per strain and time point. Values represent averages from three independent biological replicates (*n* = 3) and the error bars represent the standard deviation. Statistical analyses were performed by Student's *t* test, and significant differences are indicated. *p < 0.05 and **p < 0.005.

Arsenic has been shown to induce protein misfolding and aggregation (Jacobson et al., 2012; Tamás et al., 2014) and the protein aggregation levels can be used as a measure of intracellular damage caused by arsenic (Andersson et al., 2021; Jacobson et al., 2012; Talemi et al., 2014). To assess protein aggregation in vivo, we monitored and quantified the subcellular distribution of GFP‐tagged Hsp104, a molecular chaperone that associates with misfolded and aggregated proteins during As(III) stress (Jacobson et al., 2012). Plasmids overexpressing *PHO86*, *TUL1*, *VBA3*, or *UGP1* were introduced into BY4741 cells harboring *HSP104*‐*GFP* integrated into the genome, the transformants exposed to 2 mM As(V) and the fraction of cells containing Hsp104‐GFP foci/protein aggregates were quantified. As shown in Figure 4, 60%–70% of the cells had protein aggregates/Hsp104‐GFP foci after 1 h of As(V) exposure. The fraction of cells with aggregates/Hsp104‐GFP foci declined during the time‐course of the experiment. Interestingly, *UGP1* overexpressing cells had significantly less aggregates after 3 h (*p* = 0.0002) and 5 h (*p* = 0.02) of As(V) exposure than cells carrying the empty vector. This is in line with the observation above that *UGP1* overexpressing cells accumulate less intracellular arsenic (Figure 3). *PHO86* and *TUL1* overexpressing cells had similar levels of aggregated proteins as control cells carrying the empty plasmid (Figure 4). Thus, the increased intracellular arsenic measured in *PHO86* overexpressing cells does not seem to cause enhanced protein aggregation. Cells overexpressing *VBA3* contained more protein aggregates at the 3 h (*p* = 0.004) and 5 h time points (*p* = 0.0002) than control cells (Figure 4). Thus, the increased intracellular arsenic levels in *VBA3* overexpressing cells (Figure 3) appear to enhance protein misfolding/damage.

### 3.6 *PHO86*overexpression does not further improve As(V) resistance in mutants lacking specific phosphate transporters

**Figure 5:**
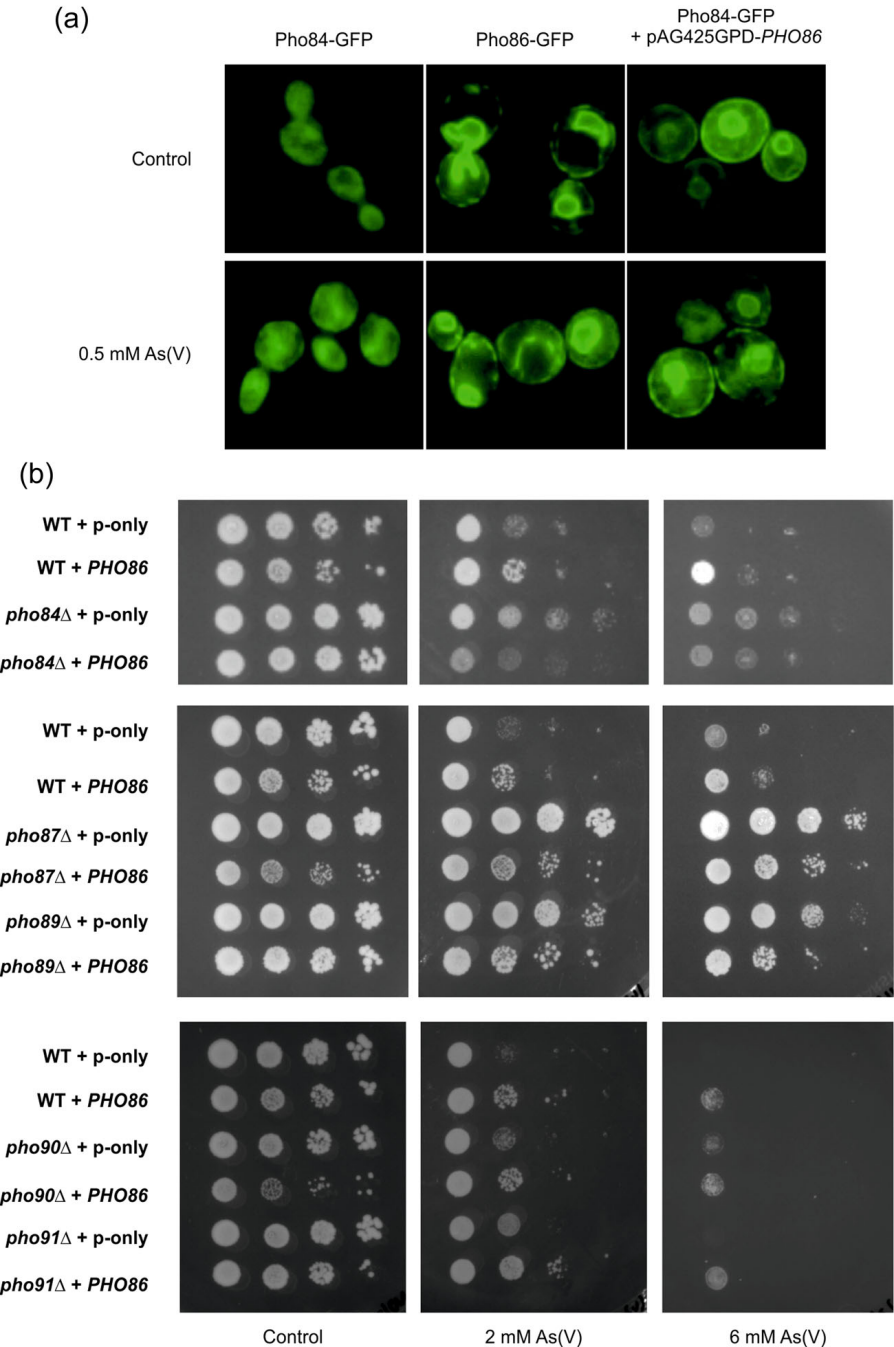
*PHO86*overexpression and phosphate transporters. (a) Localization of Pho84‐GFP in BY4741 cells overexpressing *PHO86* and exposed to As(V). (b) Growth of BY4741 cells (wild‐type and mutants lacking individual phosphate transporters) overexpressing *PHO86* in the presence of As(V). Growth was scored after incubating the cells for 2–3 days at 30°C. P‐only represents the empty vector containing cells.

The high‐affinity phosphate transporter Pho84 is known to play a role in As(V) import; its overexpression causes over‐accumulation of As(V) in the cell (Shen et al., 2012), and *PHO84* deletion results in increased As(V) resistance (Bun‐ya et al., 1996). Pho86 is required for the exit of Pho84 from the ER to the plasma membrane. In a *PHO86* deletion mutant, Pho84 is unable to localize to the plasma membrane (Lau et al., 2000) and *pho86Δ* cells are As(V) resistant (Bun‐ya et al., 1996). To address whether Pho84 localization is affected by *PHO86* overexpression, we visualized the GFP‐fused Pho84 protein in the absence and presence of As(V). We also visualized the GFP‐fused Pho86 protein. As reported earlier (Lau et al., 2000), Pho84‐GFP is localized to the plasma membrane while Pho86‐GFP is localized to the ER (Figure 5a). The presence of As(V) seemed to influence the localization of neither Pho84‐GFP nor Pho86‐GFP. In *PHO86* overexpressing cells, the overall signal intensity of Pho84‐GFP was increased and Pho84‐GFP was clearly visible in internal structures, likely the ER, and on the cell surface (Figure 5a). Thus, more Pho84 appears to be present on the plasma membrane when *PHO86* is overexpressed. This is consistent with the observation that *PHO86* overexpressing cells have enhanced intracellular arsenic levels (Figure 3). Because of the close functional relationship between Pho86 and Pho84, we asked whether Pho84 is needed for *PHO86* overexpression to confer As(V) resistance. In contrast to the effect in wild‐type cells, overexpression of *PHO86* in the *pho84*Δ mutant did not result in an additional gain of As(V) resistance (Figure 5b). The *S. cerevisiae* genome encodes five phosphate transporters and we overexpressed *PHO86* in mutants lacking the high‐affinity transporter *PHO89* and the low‐affinity transporters *PHO87*, *PHO90*, and *PHO91* (Wykoff & O'Shea, 2001) and scored growth of the transformants. Cells lacking *PHO87* and *PHO89* were more As(V) resistant than wild‐type or *pho84*Δ cells carrying an empty plasmid (Figure 5b). Thus, in addition to Pho84, Pho87 and Pho89 might be physiologically relevant As(V) import routes in yeast. The growth of *pho90*Δ and *pho91*Δ was similar to wild‐type cells in the presence of As(V). *PHO86* overexpression enhanced As(V) resistance of the *pho90*Δ and *pho91*Δ mutants but not of the arsenate‐resistant *pho87*Δ and *pho89*Δ mutants (Figure 5b). We conclude that *PHO86* overexpression does not further improve As(V) resistance in mutants lacking specific phosphate transporters.

## 4 DISCUSSION

The yeast genome‐wide deletion collection has provided important insights into arsenic toxicity and resistance mechanisms (Haugen et al., 2004; Jin et al., 2008; Jo et al., 2009; Johnson et al., 2016; Pan et al., 2010; Thorsen et al., 2009; Zhou et al., 2009). In contrast, genome‐wide overexpression to identify arsenic resistance genes has, to our knowledge, only been performed once; that screen identified the arsenic resistance locus containing *ARR1/ACR1/YAP8*, *ARR2/ACR2*, and *ARR3/ACR3* (Bobrowicz et al., 1997) showing the power of the overexpression approach. In this current study, we screened a yeast genomic library and identified novel genes, namely *PHO86*, *TUL1*, *VBA3, UGP1*, and *ERG6* that provide resistance to arsenicals in yeast cells when overexpressed. Our screen also identified known arsenic resistance‐related genes, including *ARR1/ACR1/YAP8*, *ARR2/ACR2, ARR3/ACR3*, and *YAP1*. Some of the genes in the cassettes did not enhance resistance when overexpressed individually (*YJL118W, AIM26, MTC1, ASF1, NCA3, RPE1, ALB1, LSM1*). Individual overexpression of some genes (*SGE1, YPR196W, GIS4, MRPL39*) was not tested.

*PHO86*overexpression conferred resistance to As(V) in wild‐type and arsenic‐sensitive GK4 cells but did not result in As(III) resistance, suggesting an As(V)‐specific function of Pho86 in resistance. Pho86 is an ER‐resident protein that is necessary for the exit of Pho84 from the ER into COPII vesicles (Lau et al., 2000). Pho84 is a phosphate transporter that also mediates arsenate import into cells (Shen et al., 2012). Pho84 is not properly delivered to the plasma membrane in *pho86Δ* cells (Lau et al., 2000) leading to As(V) resistance (Bun‐ya et al., 1996). Using a Pho84‐GFP construct, we found that *PHO86* overexpression resulted in increased levels of Pho84 at the plasma membrane. Additionally, *PHO86* overexpressing cells had elevated levels of intracellular arsenic, consistent with a higher As(V) uptake capacity, likely mediated by Pho84. Indeed, overexpression of *PHO84* has previously been shown to result in higher intracellular accumulation of arsenic in the cell (Shen et al., 2012). Despite this, *PHO86* overexpressing cells did not have elevated intracellular damage, as determined by protein aggregation assays. These observations suggest that *PHO86* overexpressing cells efficiently protect the proteome from intracellular arsenic, preventing its toxic effects on protein folding and activity. In addition to *pho84*Δ, we found that *pho87*Δ and *pho89*Δ cells were As(V)‐resistant, suggesting that Pho87 and Pho89 are physiologically relevant As(V) import routes. *PHO86* overexpression did not further enhance resistance of the arsenate‐resistant *pho84*Δ, *pho87*Δ, and *pho89*Δ mutants, suggesting that *PHO86*‐mediated resistance is particularly relevant when cells accumulate high intracellular As(V). Indeed, we observed a substantial growth improvement upon *PHO86* overexpression of the As(V)‐sensitive strain GK4 that lacks arsenate reductase activity and capacity to convert intracellular As(V) to As(III). How does *PHO86* overexpression confer As(V) resistance despite higher intracellular arsenic levels? The yeast vacuole is a major storage compartment for phosphate and the vacuolar membrane contains proteins that mediate phosphate and As(V) transport (Booth & Guidotti, 1997). Our data indicate that *PHO86* overexpressing cells may not sequester large amounts of As(V) in the vacuoles since these cells exported arsenic as fast as control cells upon removal of extracellular As(V). Instead, we hypothesize that higher levels of intracellular phosphate may counteract the toxic effects of As(V) as increased amounts of Pho84 at the plasma membrane caused by *PHO86* overexpression not only results in increased As(V) uptake but also in increased phosphate uptake (Rothstein, 1963). Nevertheless, we cannot rule out other possible mechanisms. For example, similar to our observations, *PHO84* overexpression was reported to confer resistance to Mg^2+^, Cu^2+^, and Co^2+^ despite higher levels of intracellular metal accumulation. The same study showed that ectopic *PHO84* overexpression activated the unfolded protein response (UPR) pathway (Ofiteru et al., 2012). Thus, increased levels of Pho84, as observed in *PHO86* overexpressing cells, might result in UPR activation that in turn mitigates metal toxicity. Alternatively, As(V) can induce iron deficiency by triggering the degradation of Fet3, a protein required for high‐affinity iron uptake in yeast (Batista‐Nascimento et al., 2013). Interestingly, Pho86 was shown to interact with Fet3 in a large‐scale screen of membrane protein interactors (Miller et al., 2005) raising the possibility that *PHO86* overexpression alleviates As(V) toxicity by enhancing iron uptake. Nevertheless, the exact mechanism by which *PHO86* overexpression results in improved As(V) resistance remains to be elucidated.

*VBA3*overexpression conferred resistance to both As(V) and As(III) and the effect was particularly pronounced in GK4 cells lacking the *ARR/ACR* gene cluster. *VBA3* encodes a basic amino acid transporter that mediates the influx of lysine and histidine into vacuoles (Shimazu et al., 2005). *VBA3* overexpressing cells had elevated intracellular arsenic content and protein misfolding/damage, yet these cells were As(V) resistant. One way this could be achieved is by Vba3‐mediated transport of arsenic into the vacuole. However, *VBA3* overexpressing cells were more efficient in exporting arsenic than control cells once extracellular As(V) was removed, arguing against a function of Vba3 in vacuolar arsenic sequestration. We have previously shown that cells increase their protein degradation capacity during arsenic exposure to mitigate toxicity (Jacobson et al., 2012). It is reasonable to assume that there is a concomitant increase in amino acid uptake into vacuoles to maintain cytosolic concentrations of amino acids at appropriate levels during arsenic stress since high intracellular amino acid levels are toxic to cells (Hughes & Gottschling, 2012; Hughes et al., 2020; Ruiz et al., 2020). Whether *VBA3* overexpression confers As(V) resistance through an increased capacity to transport amino acids into the vacuole or through another mechanism remains to be established.

*UGP1*overexpression conferred As(V) resistance. Ugp1 catalyzes the reversible formation of UDP‐glucose from glucose 1‐phosphate and UTP, and the protein is involved in a variety of metabolic pathways. *UGP1* overexpression resulted in a modest decrease in intracellular arsenic and less damage as measured by protein aggregation. Elevated resistance in *UGP1* overexpressing cells might thus be caused by this reduction in intracellular arsenic. The mechanisms by which Ugp1 affects arsenic uptake or efflux remain unknown.

*TUL1*overexpression conferred As(V) resistance without affecting intracellular arsenic or protein aggregation levels. Tul1 is a ubiquitin ligase required to sort membrane proteins into multivesicular bodies for further delivery to the vacuole (Reggiori & Pelham, 2002). Interestingly, *TUL1* overexpression has been shown to induce the degradation of several vacuolar membrane proteins, including Ycf1 (Li et al., 2015). Whether Tul1 contributes to As(V) resistance through ubiquitination and degradation of these proteins or other mechanisms remains to be unveiled.

To sum up, this current study took advantage of a yeast genome library to identify genes that contribute to arsenic resistance upon overexpression. Some of the overexpressed genes had an impact on intracellular arsenic and protein aggregation levels giving clues on how these genes confer resistance. However, dedicated follow‐up experiments will be required to provide insights into the underlying mechanisms.

## AUTHOR CONTRIBUTIONS

**Esin Isik:** investigation; formal analysis; writing–original draft preparation. **Çiğdem Balkan:** investigation; formal analysis. **Vivien Karl:** investigation; formal analysis. **Hüseyin Çağlar Karakaya:** investigation; formal analysis. **Sansan Hua:** investigation; formal analysis. **Sebastien Rauch:** investigation; formal analysis. **Markus J. Tamás:** conceptualization; formal analysis; funding acquisition; writing–original draft preparation; writing–review and editing. **Ahmet Koc:** conceptualization; formal analysis; writing–original draft preparation; writing–review and editing.

## CONFLICTS OF INTEREST

None declared.

## ETHICS STATEMENT

None required.

## Data Availability

All data generated or analyzed during this study are included in this published article.

## 1

See Table A1 and Figures A1, A2, A3, A4.

**Table A1:** *Saccharomyces cerevisiae* strains used in this study

Strain	Genotype	Source
BY4741	*MATa, his3Δ1 leu2Δ0 met15Δ0 ura3Δ0*	EUROSCARF
BY4743	*MATa/α his3Δ1/his3Δ1 leu2Δ0/leu2Δ0*	EUROSCARF
	*LYS2/lys2Δ0 met15Δ0/MET15 ura3Δ0/ura3Δ0*	
(n) Δgene	BY4741 ΔDeleted gene: KAN^R^	EUROSCARF
(2n) Δgene	BY4743 ΔDeleted gene: KAN^R^	EUROSCARF
W303‐1A	*MATa ura3–1 leu2–3/112 trp1–1 his3–11/15 ade2–1 can1–100 GAL SUC2 mal0*	Thomas and Rothstein (1989)^b^
_GK4_ a	W303‐1A *acr1Δ acr2Δ acr3Δ::loxP‐kanMX‐loxP*	Robert Wysocki
HSP104‐GFP	BY4741 *HSP104‐GFP‐HIS3‐MX6*	Invitrogen

^a^ *ACR1* is also called *ARR1*/*YAP8*; *ACR2* is also called *ARR2*; *ACR3* is also called *ARR3*.

^b^ Thomas and Rothstein (1989).
